# Effects of Fusu mixture (Wen-Shen-Qian-Yang Method) on sepsis-induced acute respiratory distress syndrome

**DOI:** 10.1097/MD.0000000000021066

**Published:** 2020-07-17

**Authors:** Li Zhang, Kunlan Long, Chunxia Wang, Xuemei Zhang, Hongjing Yang, Jun Chen, Xue Li, Peiyang Gao, Song Zhang

**Affiliations:** a Department of Critical Care Medicine; b Department of Respiratory Medicine, Hospital of Chengdu University of Traditional Chinese Medicine, Chengdu, China.

**Keywords:** acute respiratory distress syndrome, clinical trial, effects, fusu mixture, randomized control, sepsis, traditional Chinese medicine

## Abstract

**Introduction:**

Sepsis is the most common etiology of acute lung injury (ALI) or acute respiratory distress syndrome (ARDS). Capillary leakage caused by lung endothelial injury is the central cause of ARDS. The results of research in modern medicine in reducing endothelial damage and restoring endothelial functions are limited. In the previous clinical observations, we found that the Fusu mixture not only improves the clinical symptoms but also reduces the leakage of pulmonary capillaries. Therefore, the purpose of this study is to determine the clinical efficacy of the Fusu mixture combined with Western medicine in the treatment of ARDS caused by sepsis and to explore the mechanism of traditional Chinese medicine.

**Methods:**

This is a prospective, single-center, randomized, single-blind, and controlled clinical study involving 620 eligible patients. The patients will be randomly divided into 2 groups: the Western medicine treatment group and the combination of Chinese and Western medicine treatment group. After 14 days of intervention, the clinical efficacy and safety of the Fusu mixture on sepsis-induced ARDS patients will be observed. The primary outcome will be measured as 28-day mortality. The secondary outcome indices include inflammatory markers (CRP, PCT, IL-6, TNF - α), APACHE II score, SOFA score, days without a ventilator, blood gas analysis (Lac, PaO2 / FiO2), intensive care unit hospital stay time, intensive care unit mortality. Simultaneously, the analysis of the exploratory results will be carried out to analyze the possible mechanism of Fusu mixture in the treatment of sepsis-induced ARDS by the high-throughput sequencing and bioinformatics.

**Discussion:**

The purpose of this study is to evaluate the clinical efficacy of Fusu mixture in the treatment of sepsis-induced ARDS and explore its possible mechanism of action. If successful, it will provide evidence-based adjuvant therapy for the clinical treatment of ARDS.

## Abstract 2

Supplemental Digital Content is available in the text

## 1 Introduction

Sepsis is a serious life-threatening inflammatory reaction caused by infection. The pathological process involves multiple organs. The lung is the most vulnerable target organ for sepsis, and ALI/ acute respiratory distress syndrome (ARDS) appears at the earliest and has the highest incidence.^[1]^ Presently, with no definitive treatment, sepsis-induced ARDS is mainly managed by symptomatic and supportive treatment, and its morbidity and mortality are still high. 25% to 50% of patients with severe sepsis are complicated with ALI/ARDS.^[2]^ The development of ALI/ARDS can significantly worsen the prognosis of patients, causing the mortality rate of septic shock in the intensive care unit (ICU) to rise from 11% to 38% with the mortality rate as high as 40% in some patients with ALI/ARDS.^[3]^ Most of the deaths were attributed to sepsis and multiple organ failure and not to primary respiratory failure. Therefore, there is an urgent need to carry out more extensive and in-depth research on the pathogenesis, prevention, and treatment strategies of ARDS.

The functional change of pulmonary vascular endothelial cells is a key link in the pathophysiological process of sepsis-induced ARDS, which plays an important role in microcirculation abnormality, septic shock, and multi-organ dysfunction. Vascular endothelial cells are highly bioactive and participate in the regulation of vascular smooth muscle tone, the exchange of cells and nutrients, the maintenance of blood flow, and the balance of local pro-inflammatory and anti-inflammatory mechanisms.^[4]^ Basic experiments show that inflammation-induced capillary leakage occupies an important position in the pathogenesis of ARDS. Inflammation factor stimulation leads to lung endothelial cell damage or apoptosis, and endothelial barrier damage, leading to an increase in the vascular endothelial permeability and an increase in the liquid infiltration tissue clearance,^[5,6]^ which belongs to the traditional Chinese medicine (TCM) in the category of "water drink syndrome”.

The pathogenesis of the disease is Yang deficiency and water overflowing, which cannot be attributed to the ordinary way, resulting in tissue edema, and kidney yang is the root of the whole body Yang Qi. Therefore, we use the Fusu mixture to treat sepsis-induced ARDS patients, which makes the kidney yang warm and latent in the lower energizer. The Fusu mixture is composed of 6 TCMs, namely, Aconiti Lateralis Radix Praeparata, Carapax Testudinis, Amomi Fructus, Ephedra sinica Stapf, Rhizoma zingiberis, and Radix glycyrrhizae.

TCM has a long history and rich experience in the treatment of infectious diseases. At the early stage, we determined the main components of the Fusu mixture by HPLC and found that the compound contained glycyrrhizin, glycyrrhizic acid, 6-gingerol, and other components. Through animal experiments, it was confirmed that the TCM resuscitation mixture could improve the pathological changes of lung tissue in rats, inhibit the expression of heparanase in the plasma of acute lung injury caused by sepsis, and reduce the release of inflammatory factors. This shows that the Fusu mixture has a certain effect on sepsis-induced ARDS,^[7]^ which provides basic evidence for the clinical treatment of the Fusu mixture.

In the previous clinical practice, we found that the TCM Fusu mixture could supplement the basic treatment of Western medicine, clinically reduce the Extravascular lung water index of patients with sepsis-induced ARDS, and improve the prognosis of patients. ^[8]^ However, more sufficient clinical evidence is needed to confirm this. Therefore, our goal is to conduct a prospective, single-center, single-blind, randomized controlled trial to evaluate the efficacy and safety of Fusu mixture in the treatment of sepsis-induced ARDS. The results of this trial may provide evidence that the Fusu mixture is an effective prescription for sepsis leading to ARDS.

Current studies have shown that miRNA, circRNA, and lncRNA are related to endothelial barrier function, ^[9–18]^ while the regulatory mechanism of the complex regulatory network based on the whole transcriptome on the endothelial function of sepsis has not been reported. Therefore, based on these previous studies, we hypothesize that the Fusu mixture may achieve therapeutic effects by regulating the expression of genes in the whole transcriptome.

## 2 Method and design

### 2.1 Design

**Figure 1:**
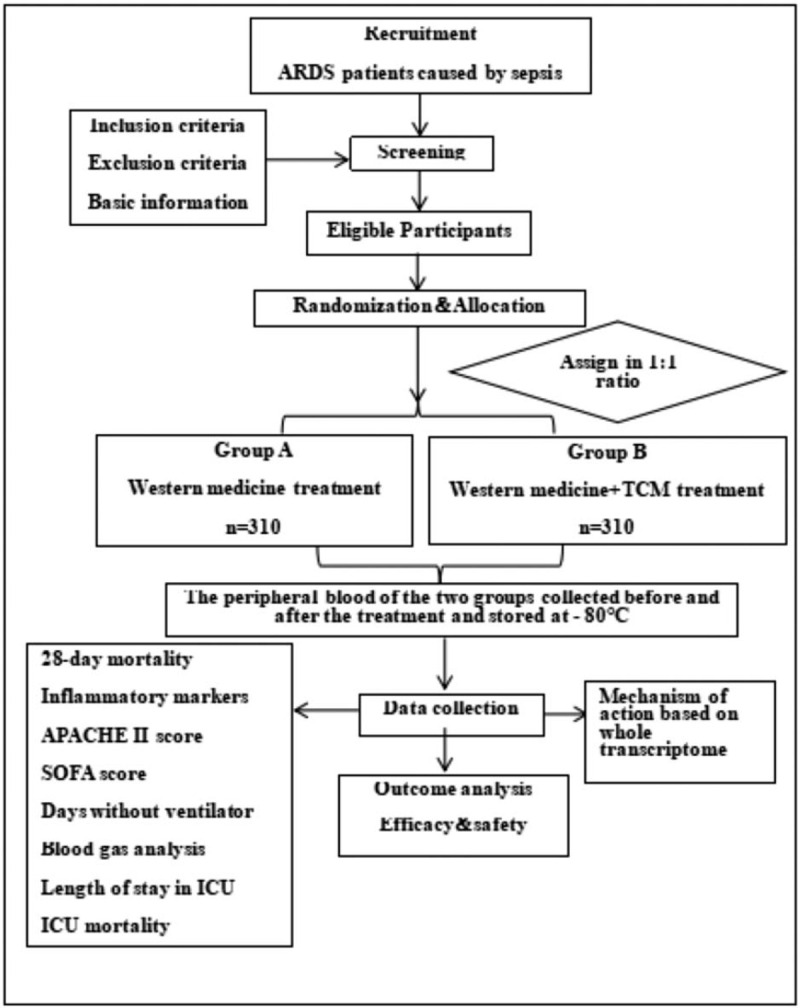
Flow chart of the study design. ARDS = acute respiratory distress syndrome, TCM = traditional Chinese medicine.

**Figure 2:**
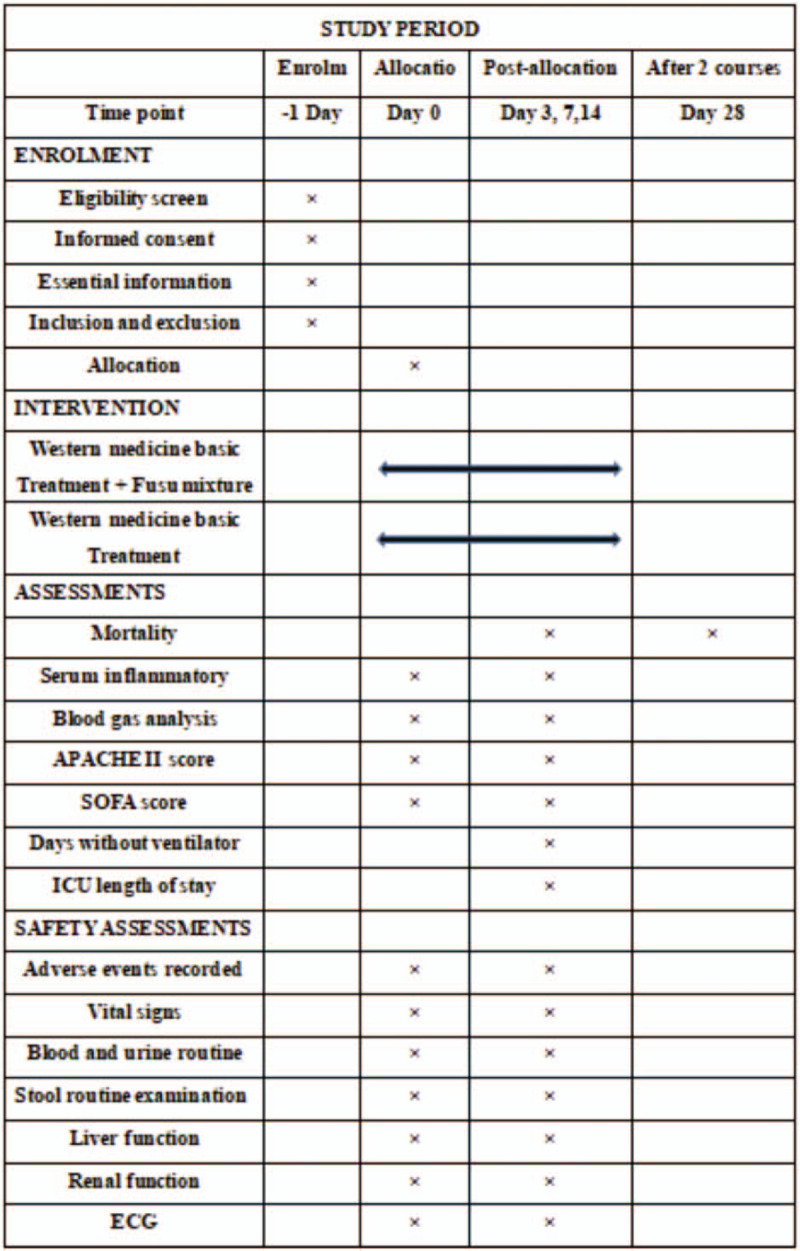
Schedule of enrollment, interventions, assessments, and data collection. CRP = Serum inflammatory index includes, PCT, IL-6, TNF-a; blood gas analysis includes: Lac, PaO2/FiO2; Liver function index monitoring includes: ALT, AST, Tbil, GGT, ALP; Renal function index monitoring includes: Scr, BUN, eGFR. ALP = alkaline phosphatase, ALT = alanine aminotransferase, AST = aspartate aminotransferase, BUN = blood urea nitrogen, CRP = C-reactive protein, eGFR = estimated glomerular filtration rate, GGT = γ-glutamyl-transferase, IL-6 = interleukin-6, Lac = lactic acid, PaO2/FiO2 = oxygenation index, PCT = procalcitonin, Scr = serum creatinine, Tbil = total bilirubin, TNF-α = tumor necrosis factor-alpha.

This is a single-center, single-blind, randomized controlled trial involving 620 patients who will be randomly assigned to either the Western medicine treatment group or the integrated Chinese and Western medicine treatment group. Each treatment course is for 7 days, which includes 2 courses of treatment. The clinical efficacy and safety of Fusu mixture for sepsis-induced ARDS patients will be evaluated, and the related mechanisms of action will be discussed based on the whole transcriptome sequencing. This study will adhere to the Standard Protocal Items: Recommendation for Interventional Trials 2013 statement. Refer to Document 1, for the Standard Protocol Items: Recommendations for Interventional Trials checklist.^[19]^ The study flow chart is shown in Figure 1 and the specific timing of the study and the collection of data from baseline to follow-up are shown in Figure 2.

### 2.2 Moral certification

The study was approved after meeting the requirements set by the ethics committee of the affiliated hospital of Chengdu University of TCM (No. 2019KL-049). Following the Declaration of Helsinki (Edinburgh, 2000), this study trial (ChiCTR1900027988) was registered with clinicaltrials.gov on December 7, 2019 (Version. V1.0), and all participating staff was trained in standard operating procedures. If any amendments are made to the agreement, the review and approval of the ethics committee will be sought again.

### 2.3 Research environment and participants

All patients with sepsis-induced ARDS admitted to the general and respiratory ICU of the affiliated hospital of Chengdu University of TCM during the study period will be screened and enrolled. Informed consent will be obtained from eligible patients and then they will be randomly divided into the Western medicine treatment group and the integrated Chinese and Western medicine treatment group in a 1:1 ratio. If the patient is unable to give informed consent at the time of admission to ICU due to the severity of ARDS, informed consent will be obtained from the next of kin or from an independent physician. In these cases, we will obtain informed consent from the patient as soon as possible.

### 2.4 Sample size

Calculating the sample size was based on the 28-day mortality. According to the available literature, the 28-day mortality of the western medicine treatment group is about 40%, whereas that of the treatment group with integrated traditional Chinese and Western medicine is about 30%. The software Power Analysis and Sample Size version 11.0 (PASS 11.0) was used to calculate the sample size of the 2 groups, assuming that the test level is *α* = 0.05, the probability of making mistakes *β* = 0.2, considering the 10% loss rate, a total of 620 patients will be enrolled.

### 2.5 Randomization and allocation concealment

Members of The Sichuan TCM Evidence-Based Medicine center will use SAS 9.2 software (SAS, Cary, NC) to generate 620 random serial numbers. After screening and baseline evaluation, the patients with ARDS will be randomly divided into 1 of the 2 groups. Eligible patients will be assigned to the treatment group of integrated traditional Chinese and Western medicine or the treatment group of Western medicine alone at a proportion of 1:1. The group number will be provided in a carbon-free sealed envelope. The envelope will be kept by the study administrator, who will not be directly involved in any participant's recruitment or follow-up, and the group number will be disclosed later. The administrator will open an envelope and provide participants with their group number on the day of inclusion.

### 2.6 Diagnostic criteria

Participants must meet the diagnosis criteria for both sepsis and ARDS. For the diagnosis standard of sepsis, please refer to the “Sepsis3,0 Diagnosis criteria which was published by the American Medical Association in 2016,”^[20]^ and the “2018 emergency treatment guidelines for sepsis/septic shock in China”.^[21]^ For patients with infection or suspected infection, sepsis can be diagnosed when the score of sepsis related sequential organ failure (sofa) is more than or equal to 2 points higher than the baseline. See Table 1 for the scoring criteria of sofa and ARDS Berlin diagnostic criteria are shown in Table 2.^[22]^

**Table 1:**
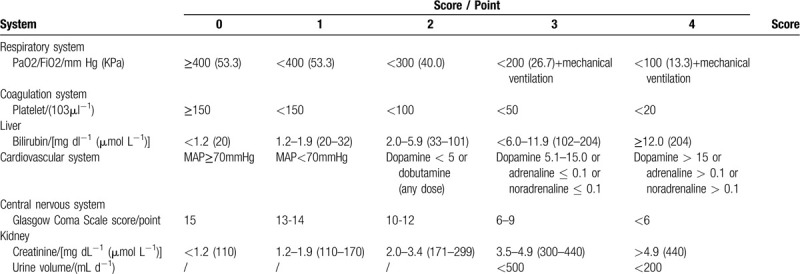
SOFA scoring standard: the dosage of catecholamine isμg/(kg min), the dosage is at least 1 h, and the score range of Glasgow coma scale is 3 to 15 points. The higher the score, the better the neurological function.

**Table 2:**
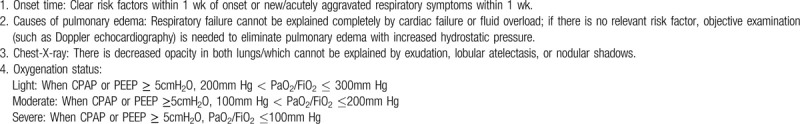
Western medicine diagnostic criteria for acute respiratory distress syndrome.

### 2.7 Qualification criteria

#### 2.7.1 Inclusion criteria

Patient must meet all the following criteria:

(1) Patients 18 to 80 years of age.
(2) The course of the disease within 12–48 hours, meeting the diagnostic criteria for sepsis and ARDS.
(3) Oxygenation index (PaO2/FiO2) ≤200 in ARDS.
(4) Patients with mechanical ventilation.
(5) In order to conform to the Declaration of Helsinki and Chinese clinical trial research regulations, the patient or family members must know the content of the study and sign informed consent voluntarily.

#### 2.7.2 Exclusion criteria

Patients who meet 1 or more of the following exclusion criteria cannot be included:

(1) Pregnant or lactating women.
(2) Patients allergic to the drugs used in the trial.
(3) Those with indications of surgery.
(4) Patients with other serious physical diseases, AIDS, and malignant tumors.
(5) Patients who are expected to die or give up an active rescue or refuse conventional supportive treatment in Western medicine within 12 hours of admission to the ICU.
(6) Patients with contraindications for femoral artery catheterization, central venous catheter placement, and blood transfusion.
(7) Patients with chronic heart failure or end-stage multi-organ failure.
(8) Currently participating in other pharmaceutical research.
(9) Patients who are employees of the research site or family members of employees at the research site.

### 2.8 Termination and withdrawal criteria

All participants will be informed that they have the right to withdraw from the trial and if they do, they will also receive the standard treatment. The reason for withdrawal will be recorded in their case report file (CRF). The criteria for discontinuing treatment and withdrawing patients from the study are:

(1) The experience of adverse events related to taking the drugs, and the researchers determine that it is not appropriate to continue administering the drugs
(2) Required treatment for another serious disease during the study
(3) Poor compliance or withdrawal midway through the study
(4) Inability to tolerate 2 weeks of the intervention and cannot continue the research due to the automatic requirement of discharge or transfer
(5) Giving up treatment in advance due to economic burden and/or other factors
(6) The experience of a serious adverse event in the process of the trial, such as a life-threatening incident or even death, and the researchers could not continue

### 2.9 Test drugs

Fusu mixture is a compound preparation composed of TCM. The main composition is shown in Table 3. Which was provided by the Chengdu University of TCM affiliated Hospital Pharmacy Department. The specific preparation method is to decoct the Baifu tablet with water for 1.5 hour and add the other 5 drugs. Add 6 times water for 50 minutes, decoct 3 times. Concentrate the filtrate to the relative density of 1:1.1, add 0.2% benzoic acid, sub pack to 100 ml per bottle.

**Table 3:**
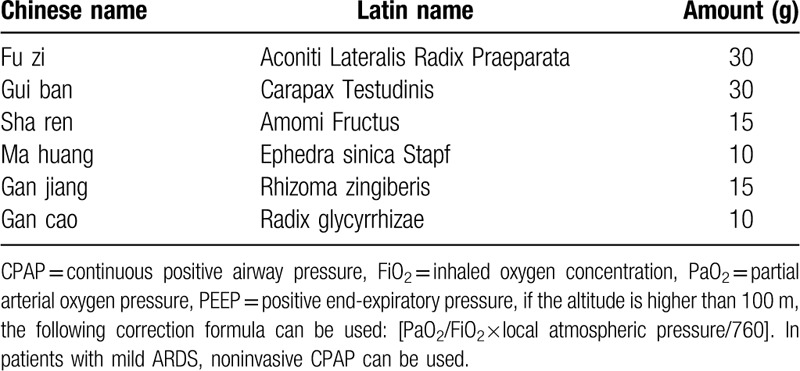
Main components of Fusu mixture.

## 3 Interventions

### 3.1 Treatment plan

#### 3.1.1 Western medicine treatment group

Participants will receive standard western medicine treatment in accordance with the “2018 emergency treatment guidelines for sepsis/septic shock in China” and the “diagnosis and treatment guidelines for acute lung injury/acute respiratory distress syndrome”(revised in 2006)^[23]^ Including early quantitative liquid resuscitation, liquid management, and nutrition support; blood culture before starting antibiotics; imaging examination to find potential infection focus; boosting pressure, strengthening heart, improving circulation and perfusion; continuous blood pressure monitoring, anti-inflammatory, and anti-shock; active control of primary disease, mechanical ventilation, maintaining blood sugar, acid-base, and electrolyte balance; maintaining normal blood clotting.

#### 3.1.2 Combined traditional Chinese and Western medicine treatment group

Participants added the Fusu mixture based on Western medicine treatment. Administering method: Each time nasal or oral administration of 25 mL, once every 6 hours, 4 times per day, 7 days as a course of treatment, a total of 2 courses of treatment.

### 3.2 Outcome measures

#### 3.2.1 Primary outcome

The 28-day mortality will be compared between the 2 groups.

#### 3.2.2 Secondary outcomes

Changes in the following indicators will be detected before and after treatment on days 3, 7, and 14.

(1) Serum levels of inflammatory markers: Validation markers including serum CRP and PCT, IL - 6, TNF-α.
(2) Blood gas analysis: Record the arterial blood gas analysis before and after the intervention (Lac, PɑO_2_ / FiO_2_)
(3) Assessment of disease severity: The APACHE II and SOFA scoring systems before and after treatment used to compare the scores of the 2 groups. Improvement rate of APACHE II score = (post-treatment score - pre-treatment score) / pre-treatment score. The APACHE II scoring system can be used as an indicator to evaluate the condition and prognosis of ICU patients, which is composed of three parts: Acute Physiology Score (APS), age score and chronic health status score, the final score is the sum of the three parts and the highest score is 71. A higher score indicates worse disease. The SOFA score can be used to assess the occurrence and severity of organ dysfunction in critically ill patients, and the worst value of the day should be adopted for counting. The higher score indicates the worse prognosis.
(4) The number of days without ventilator: The number of days without a ventilator will be recorded from the patient's admission to ICU to the completion of 2 courses of treatment.
(5) Length of stay: The time from the patient's admission to the ICU to his/her departure from the ICU.
(6) ICU mortality: The proportion of deaths in the 2 groups in the total number of participants from the beginning of the trial to 14 days will be recorded.

#### 3.2.3 Exploratory results

The peripheral blood samples of patients will be collected at baseline and on the 7th and 14th days of treatment, and the differentially expressed genes will be analyzed by full transcriptome sequencing. Simultaneously, the expression of related factors and proteins in peripheral blood will be detected by qRT-PCR and ELISA, to explore the mechanism of the Fusu mixture.

### 3.3 Safety assessment

From baseline to the end of the study, physical examinations will be performed daily. Routine blood and urine tests, routine stool tests with occult blood, liver and renal function tests, Coagulation function and electrocardiograms will be examined at baseline and on days 3, 7, and 14, and any adverse events during the study period will be observed and recorded in detail.

### 3.4 Adverse events (AEs)

Any adverse medical event occurring in the subjects during the clinical study, regardless of whether it has a causal relationship with the study drug, is considered an AE. The AE report form will be completed during the test. Record the occurrence time, severity, duration, measures taken, and results of adverse events. If a serious adverse event occurs, it should be reported to the adverse drug reaction testing center of the local authority within 24 hours and to the sponsor at the same time.

### 3.5 Quality control and data management

Prior to the study, the protocol was reviewed and revised by clinicians, statisticians, and methodologists. All staff of the trial must participate in a series of training sessions to ensure that the involved personnel fully understood the protocol and standard operating procedures to ensure the accuracy and integrity of clinical data. The test supervisor will monitor the process of the test on a regular basis. The evaluator will first record all the data on the paper version of the CRF, and then double input it into the electronic data capture system electronically. Monitoring personnel will regularly check the completion and compliance of CRF. In order to ensure the objectivity of the data, we will ensure that observers and statisticians do not understand the data. An independent quality inspector will monitor the entire process.

### 3.6 Statistical analysis

Before analysis, 2 similar participants with complete data will be carefully examined to ensure that the data is correct. All data analyses will then be based on treatment intention.

The data will be analyzed using the statistical software package SPSS 22.0 (Chicago, IL). The analysis method will be selected according to the distribution characteristics of the data. The measured data will be expressed as the mean ± the standard deviation. First, the normal test and the homogeneity test of variance will be performed. In the case of normal distribution and equal variance, a *t*-test will be performed; otherwise, a nonparametric test will be utilized. The difference will be statistically significant when *P* ≤ .05.

### 3.7 Informed consent

Consent will be provided to patients who meet the study requirements, including the study name, research background, research method, what participants should do in the study, inclusion/exclusion criteria, treatment plan, and obligations, possible side effects of the trial drug, costs in the participation process, and so on. We will make every effort to protect the privacy of the patient's personal medical data to the extent permitted by law. Participation in this study is voluntary, and the basic Western medicine treatment program will continue for patients who do not participate or withdraw. When patients sign informed consent, their personal and medical information will be used in this study.

### 3.8 Secrecy

Participants’ medical records will be kept in hospitals and will be accessible to researchers, research institutions, and ethics committees. No public report on the results of this study will reveal the personal identity of the participants. We will make every effort to protect the privacy of the participant's personal medical data to the extent permitted by law. Personal and medical information will be kept confidential in a safe and reliable place. Participants can request access to their personal information (such as an address, contact information, etc) at any time, and can modify this information if necessary.

## 4 Discussion

Population-based data suggest that the incidence rate of ARDS is 1.5–8.3 per 100 thousand person-years, and it is likely to double in the next ten years.^[24,25]^ Currently, there is no specific drug for the treatment of ARDS. Our previous clinical observation and basic research showed that the Fusu mixture has auxiliary therapeutic effect on ARDS patients. However, so far, there is no large sample study on the efficacy and safety of Fusu mixture in the treatment of sepsis-induced ARDS.

Simultaneously, the existing literature shows that there is no report on the mechanism of Chinese medicine treating ARDS caused by sepsis based on the whole transcriptome. Therefore, it is necessary to carry out prospective tests to determine the clinical efficacy and safety of Fusu mixture and explore its mechanism.

There are limitations in this study. First, since the study was conducted in Chengdu, Sichuan Province, it is not clear whether the clinical effects of the investigational drug differ in other regions and races. Secondly, due to the limited funds and conditions, the follow-up time is short and the follow-up indicators are limited. If the trial is successful, it will provide patients and doctors with new options to treat ARDS, so as to achieve effective treatment of the disease and reduce the disease and economic burden of sepsis-induced ARDS patients and their families. In the future, multicenter randomized controlled trials should be carried out to carry out clinical research on patients with ARDS in a wide range and in multiple regions, and relevant verification should be carried out in combination with the mechanism of action.

## Acknowledgments

The authors are grateful to the National Natural Science Foundation of China (Available at: https://isisn.nsfc.gov.cn/egrantweb/) for funding this study. They also thank Editage (www.editage.cn) for English language editing.

## Author contributions

**Conceptualization:** Li Zhang, Kunlan Long.

**Data curation:** Xue Li, Hongjing Yang, Jun Chen.

**Methodology:** Li Zhang, Peiyang Gao.

**Supervision:** Peiyang Gao, Kunlan Long, Chunxia Wang, Xuemei Zhang.

**Writing – original draft:** Li Zhang, Kunlan Long.

**Writing – review & editing:** Peiyang Gao, Song Zhang.

## Supplementary Material

Supplemental Digital Content
